# Isolation and Taxonomic Characterization of Novel Haloarchaeal Isolates From Indian Solar Saltern: A Brief Review on Distribution of Bacteriorhodopsins and V-Type ATPases in Haloarchaea

**DOI:** 10.3389/fmicb.2020.554927

**Published:** 2020-12-09

**Authors:** Dipesh Kumar Verma, Chetna Chaudhary, Latika Singh, Chandni Sidhu, Busi Siddhardha, Senthil E. Prasad, Krishan Gopal Thakur

**Affiliations:** ^1^Structural Biology Laboratory, G. N. Ramachandran Protein Centre, Council of Scientific and Industrial Research-Institute of Microbial Technology (CSIR-IMTECH), Chandigarh, India; ^2^MTCC-Microbial Type Culture Collection & Gene Bank, Council of Scientific and Industrial Research Institute of Microbial Technology (CSIR-IMTECH), Chandigarh, India; ^3^Department of Microbiology, School of Life Sciences, Pondicherry University, Puducherry, India; ^4^Biochemical Engineering Research and Process Development Centre, Council of Scientific and Industrial Research-Institute of Microbial Technology (CSIR-IMTECH), Chandigarh, India

**Keywords:** haloarchaea, bacteriorhodopsin, pangenome, carotenoids, taxonomy

## Abstract

Haloarchaea inhabit high salinity environments worldwide. They are a potentially rich source of crucial biomolecules like carotenoids and industrially useful proteins. However, diversity in haloarchaea present in Indian high salinity environments is poorly studied. In the present study, we isolated 12 haloarchaeal strains from hypersaline Kottakuppam, Tamil Nadu solar saltern in India. 16S rRNA based taxonomic characterization of these isolates suggested that nine of them are novel strains that belong to genera *Haloarcula, Halomicrobium*, and *Haloferax*. Transmission electron microscopy suggests the polymorphic nature of these haloarchaeal isolates. Most of the haloarchaeal species are known to be high producers of carotenoids. We were able to isolate carotenoids from all these 12 isolates. The UV-Vis spectroscopy-based analysis suggests that bacterioruberin and lycopene are the major carotenoids produced by these isolates. Based on the visual inspection of the purified carotenoids, the isolates were classified into two broad categories i.e., yellow and orange, attributed to the differences in the ratio of bacterioruberin and lycopene as confirmed by the UV-Vis spectral analysis. Using a PCR-based screening assay, we were able to detect the presence of the bacteriorhodopsin gene (*bop*) in 11 isolates. We performed whole-genome sequencing for three *bop* positive and one *bop* negative haloarchaeal isolates. Whole-genome sequencing, followed by pan-genome analysis identified multiple unique genes involved in various biological functions. We also successfully cloned, expressed, and purified functional recombinant bacteriorhodopsin (BR) from one of the isolates using *Escherichia coli* as an expression host. BR has light-driven proton pumping activity resulting in the proton gradient across the membrane, which is utilized by V-Type ATPases to produce ATP. We analyzed the distribution of *bop* and other accessory genes involved in functional BR expression and ATP synthesis in all the representative haloarchaeal species. Our bioinformatics-based analysis of all the sequenced members of genus *Haloarcula* suggests that *bop*, if present, is usually inserted between the genes coding for B and D subunits of the V-type ATPases operon. This study provides new insights into the genomic variations in haloarchaea and reports expression of new BR variant having good expression in functional form in *E. coli*.

## Introduction

A group of microbes called extremophiles can grow, adapt, and survive harsh conditions like high salinity, high or low temperature, and acidic or alkaline conditions. Haloarchaea are extremophiles that grow in the hypersaline environments such as the natural brine, Dead Sea, alkaline salt lakes, marine solar salterns, and rock salt deposits (Cayol et al., 1994; Purdy et al., 2004; Gramain et al., 2011; Stan-Lotter and Fendrihan, 2015). Besides high salinity, haloarchaea are also exposed to very stringent conditions such as high temperature, UV radiations, high ionic stresses, and alkaline pH (Bowers and Wiegel, 2011; Stan-Lotter and Fendrihan, 2015). These microbes express specialized proteins and also produce metabolites like carotenoids that aid in adaptation, survival, and growth in such harsh environmental conditions (Giani et al., 2019). These metabolites and proteins have characteristics suitable for various industrial or research applications (Littlechild, 2015; Cabrera and Blamey, 2018; Verma et al., 2020). Haloarchaea produces carotenoids in high amounts that act as antioxidants, light protection pigments, and membrane stabilizers (Rodrigo-Banos et al., 2015; Giani et al., 2019). Bacterioruberin, a haloarchaeal carotenoid, reportedly has more free radical scavenging activity compared to plant β-carotenes, being used in various food and cosmetic products (Yatsunami et al., 2014; Higa et al., 2020).

With the depleting natural resources, different non-conventional biological materials are being explored to accomplish future energy requirements. Microorganisms such as haloarchaea with their unique features have attracted the attention of researchers to investigate them for the generation of energy (Nadella and Hernandez Baltazar, 2018). These microbes code for bacteriorhodopsin protein (BR), which has a unique property of light-driven proton-pumping activity and hence finds uses in several applications, including solar cells, optical filters, hydrogen production, biosensors, optogenetics, and memory storage devices (Bogomolni et al., 1976; Saeedi et al., 2012; Li et al., 2018). BR can be potentially used to harvest abundant solar energy to produce electricity or in the photolysis of water to generate hydrogen fuel to meet future ever-increasing energy demands (Sediroglu et al., 1998). However, the production cost of BR is one of the major bottlenecks in commercializing such innovative technologies. There are few recombinant methods available that successfully enhanced the yield of BR expression using *Escherichia coli* as an expression host (Kahaki et al., 2014; Bratanov et al., 2015; Jeganathan et al., 2019; Mirfeizollahi et al., 2019). Also, many attempts have already been made to utilize recombinant BR but until now no commercial applications have been reported. Therefore, it becomes essential to explore different saltern environments for studying new BR molecules which could essentially be utilized in biophotonics and bioelectronics applications.

There are limited studies describing microbial biodiversity in the Indian solar salterns. In one study, isolation and characterization of the *Haloarcula marismortui* RR12 strain was reported from Mumbai solar saltern, India (Thombre et al., 2016). In another study, BR purification was reported from *Haloferax larsenii* RG3D.1 strain isolated from Rocky Beach of Malvan, West Coast of India (Kanekar et al., 2015). Multiple haloarchaeal strains from Goa and Mulund solar salterns have also been reported (Rajurkar and Pathak, 2014). Our group has been working on isolation of extreme haloarchaeal strains from solar salterns to study the diversity and to identify novel BR sequences, if any, from the isolates. We reported the isolation of extremely halophilic archaea *Halogeometricum borinquense* strain wsp3 and *Haloferax volcanii* strain wsp5 from Marakkanam solar salterns Pondicherry and *Haloarcul*a strain K1^T^ from Thamaraikulam solar salterns Kanyakumari (Verma et al., 2019). In recent studies, our group has characterized several haloarchaeal strains isolated from Indian solar salterns.

In this study, we have isolated 12 haloarchaeal strains from the Kottakuppam Solar saltern, East Coast Road from Chennai to Pondicherry, Villupuram district, Tamil Nadu. The 16S rRNA gene sequencing suggested that nine of them were novel strains. We screened the strains for the presence of the bacteriorhodopsin gene (*bop*) and successfully cloned, expressed, and purified one of the recombinant BR using *E. coli* as an expression host. We also studied the genetic organization of *bop* and accessory proteins involved in the light-driven ATP synthesis from haloarchaea. We purified carotenoids from all 12 strains and performed absorption-based biophysical characterization. We further performed the comparative genomics analysis of all three *bop* positive strains, which revealed unique genes and industrially important enzymes encoded in their genomes.

## Results

### Isolation, Taxonomic Characterization, and *bop* Screening

All pws strains were isolated from different crystallizer ponds of Kottakuppam solar saltern near Puducherry, Tamil Nadu India. The different sampling locations in the crystallizer ponds yielded different haloarchaeal isolates designated as pws 1 to 12. The single colonies of pure cultures were isolated after performing 3–4 subculturing steps. Genomic DNA was isolated from the purified cultures grown in liquid broth followed by 16S rRNA amplification. The partially amplified 16S rRNA (size ranges from 500 bp to 1,200 bp) sequences were used for multiple sequence alignment. The 16S rRNA sequence similarity scores for all twelve isolates were close to 99% with the closest reference strains for pws1, pws3, pws5, pws6, pws7, pws8, pws9, pws11, and pws12 suggesting that all these nine isolates were novel strains (Table 1). EzTaxon analysis suggested that strains pws1, pws3, pws7, pws10, and pws12 belong to genus *Halomicrobium*, pws2, pws4, pws5, pws6, pws8, and pws9 belong to genus *Haloarcula* and pws11 belongs to genus *Haloferax* (Table 1). The partial 16S rRNA sequences for pws2, pws4, and pws10 shared 100% identity score with *Haloarcula salaria* JCM 15759, *Haloarcula japonica* JCM7785 and *Halomicrobium mukohataei* JP60, respectively (Table 1). All the isolates were further screened for the presence of the *bop* using degenerate primers (DegF and DegR) as reported earlier (Verma et al., 2019) (Supplementary Table 1). The presence of an expected 450 bp PCR product on an agarose gel confirmed that all the strains except pws11 were positive for *bop* (Supplementary Figure S1).

**Table 1:** 16S rRNA sequence similarity of pws isolates with the closest matches obtained using EzTaxon webserver.

**S.No**.	**Isolate**	**Strain name (Accession number)**	**Similarity**
1.	pws1	*Halomicrobium mukohataei* DSM 12286 (GCA_000023965.1)	<99%
2.	pws2	*Haloarcula salaria* JCM 15759	100%
3.	pws3	*Halomicrobium mukohataei* DSM 12286 (GCA_000023965.1)	<99%
4.	pws4	*Haloarcula japonica* JCM7785 (GCA_000336635.1)	100%
5.	pws5	*Haloarcula argentinensis DSM 12282* (GCA_000336895.1)	<99%
6.	pws6	*Haloarcula salaria* JCM 15759	<99%
7.	pws7	*Halomicrobium mukohataei* JP60 (GCA_004803735.1)	<99%
8.	pws8	*Haloarcula vallismortis* ATCC 29715	<99%
9.	pws9	*Haloarcula salaria* JCM 15759	<99%
10.	pws10	*Halomicrobium mukohataei* JP60 (GCA_004803735.1)	100%
11.	pws11	*Haloferax volcanii* DS2 (GCA_007714225.1)	<99%
12.	pws12	*Halomicrobium katesii* CECT 7257 (GCA_000379085.1)	<99%

### Morphological Characterization of pws Strains

**Figure 1:**
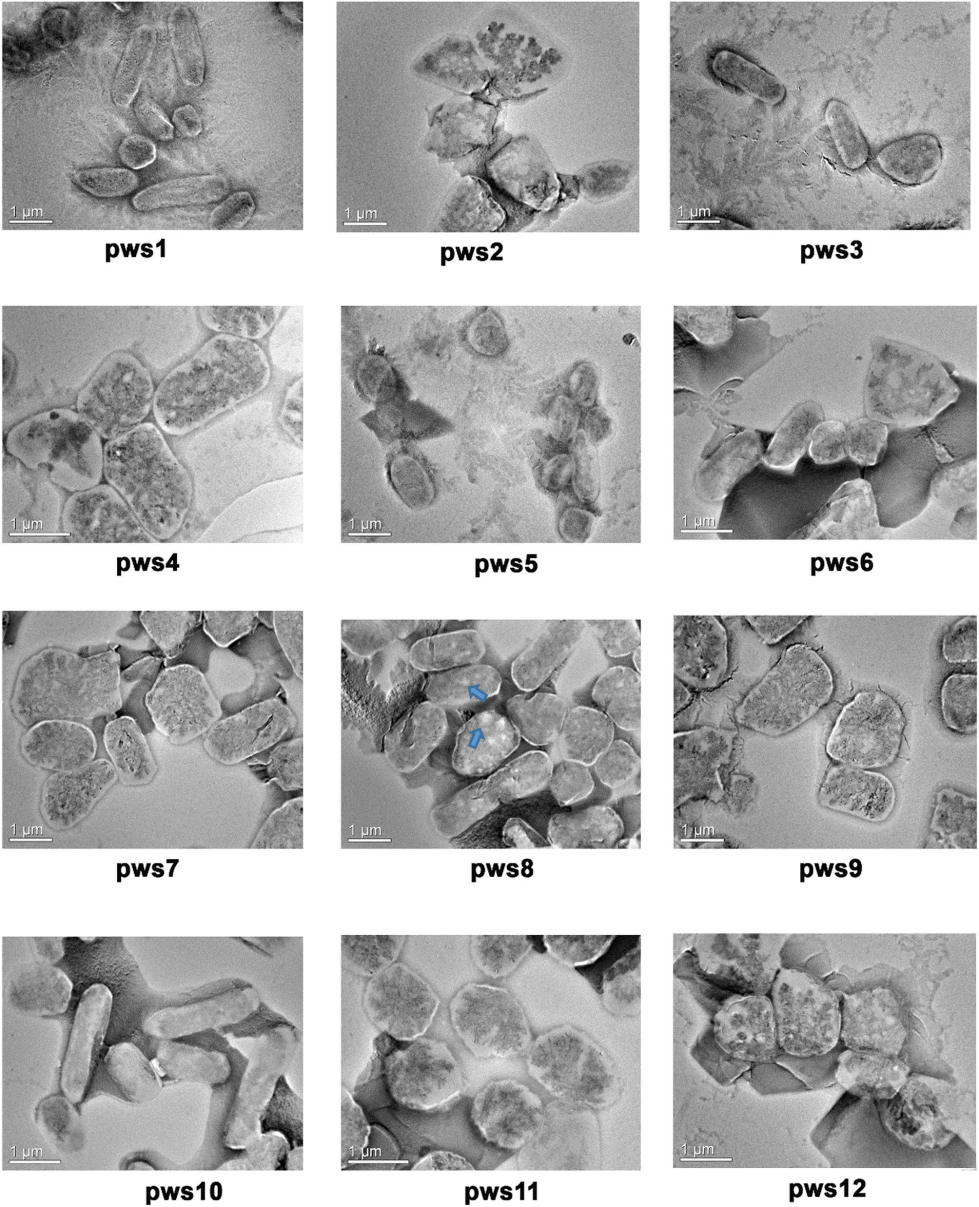
Transmission electron microscopy images of pws isolates. TEM images reveal polymorphic morphology in the haloarchaeal isolates. Blue arrows indicate the presence of gas vacuoles.

The Halobacteriaceae family members show extreme polymorphism, ranging from rods, pleomorphic rods, disc-shaped, cocci, square, and triangular forms (Fendrihan et al., 2006). The morphology feature of non-coccoid haloarchaea dependents on the salt concentration of the environment, and with decreasing salt concentration, different shapes like swollen, club-shaped, and bent rods appear (Mohr and Larsen, 1963). To visualize the cellular morphology of pws isolates, we performed transmission electron microscopy experiments (TEM). The TEM images of the isolates suggest that all strains are polymorphic, with size ranging from 0.5 to 3 μm (Figure 1). For strains pws1, pws3, and pws10, we could observe prominent rod-like morphology. TEM images of pws8 suggested that it is highly vacuolated (Figure 1). Several bacteria synthesize gas vesicles, and also few haloarchaea can produce these flotation devices (Walsby, 1994). These gas vacuoles are filled by diffusion with environmental gases dissolved in the water. The functional role of these gas vacuoles is to provide buoyancy, enabling cells to maintain their depth in the aqueous environment. Besides polymorphic morphology, we also observed some interesting features in TEM images shown in Supplementary Figure S2.

### Carotenoid Isolation and Spectroscopic Analysis

**Figure 2:**
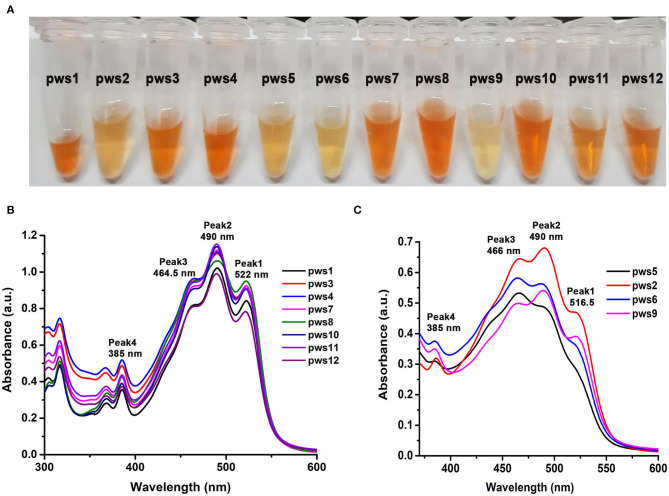
Isolation and UV-Vis absorption spectrum based characterization of carotenoids isolated from pws strains. **(A)** Carotenoids isolated from the haloarchaeal isolates using acetone extraction method. **(B)** UV-Vis absorbance profile of yellowish colored carotenoids isolated from pws1, 3, 4, 7, 8, 10, 11, and 12 samples. **(C)** UV-Vis absorbance profile of orange colored carotenoids isolated from pws5, 2, 6, and 9. Peak 1 (522 nm), peak 2 (490 nm), peak 3 (465 nm), and peak 4 (385 nm) correspond to bacterioruberin, all-trans-lycopene, 13-cis-lycopene, and all trans-β-carotene, respectively.

Haloarchaea are known to be one of the richest sources of carotenoids (Giani et al., 2019). The major component of the haloarchaeal carotenoid pool is bacterioruberin (Yatsunami et al., 2014). We isolated carotenoids from all twelve strains as described in earlier methods (Yang et al., 2015) (Figure 2A). Isolated carotenoids were grossly grouped into two distinct types, i.e., yellow and orange (Figure 2A). UV-Vis spectra of the isolated carotenoids also show differences in their composition (Figures 2B,C). In the orange samples (Figure 2B), the ratio of peak1/peak 3 is close to 1, while in the pale-yellow samples (Figure 2C), the peak1/peak3 ratio is <0.8. In standard carotenoid UV-Vis spectra, peak1 (515–522 nm) corresponds to bacterioruberin absorption; peak2 represents all-trans-lycopene absorption, and peak3 represents 13-cis-lycopene absorption (Yatsunami et al., 2014). A minor absorption of bacterioruberin is also reported at 466 nm.

Both bacterioruberin and lycopene are connected through a single pathway where lycopene is converted into bacterioruberin by lycopene cyclase. Therefore, in the second profile, the low score of peak1/peak3 ratio may suggest low bacterioruberin content (peak1) and high lycopene (peak3) accumulation in pws6, pws9, pws2, and pws5 (Figure 2C).

### The Pangenome Analysis of pws Strains

**Figure 3:**
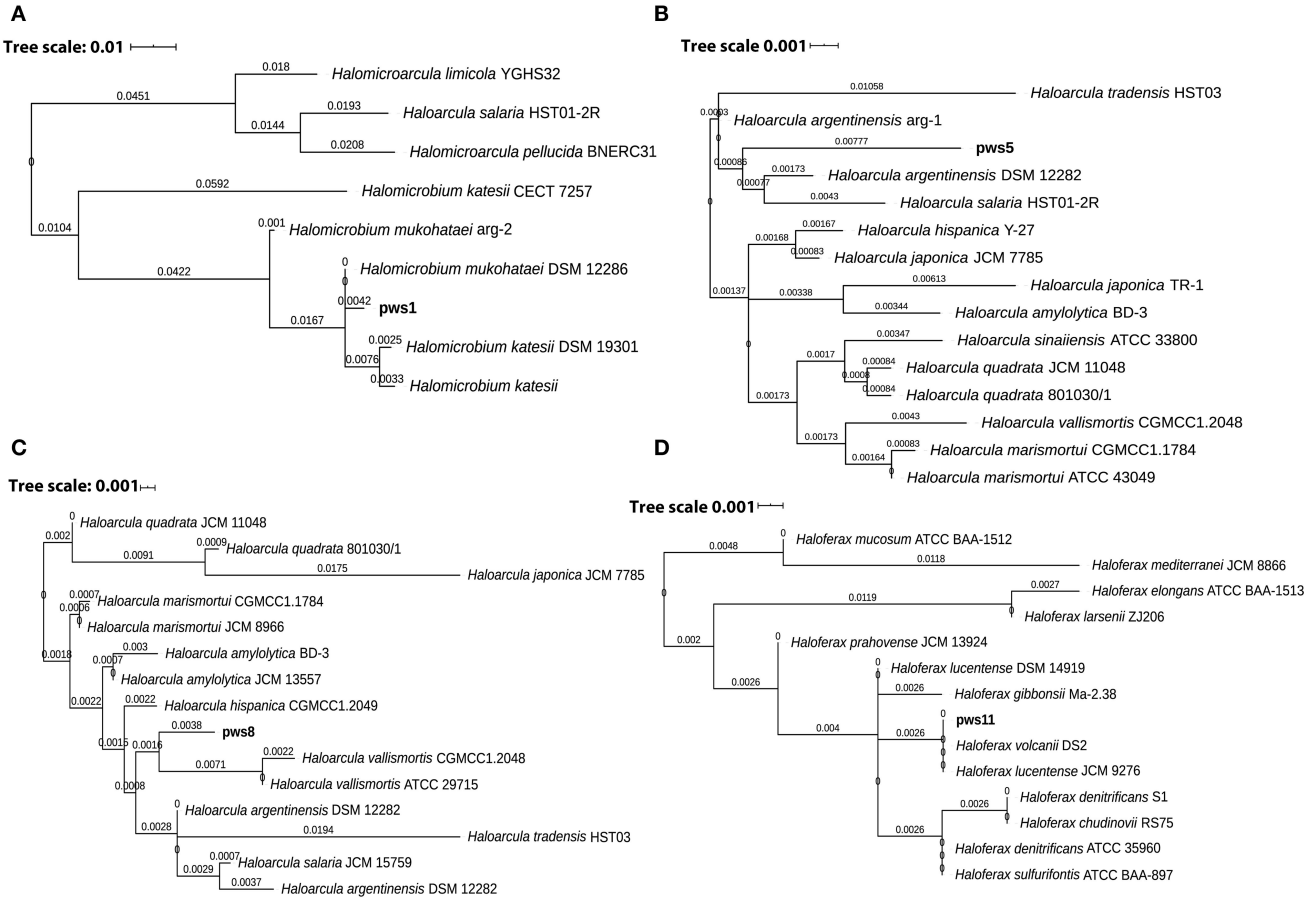
The 16S rRNA sequence-based phylogenetic tree of the isolates and the closely related haloarchaeal species. Phylogenetic analysis suggests that isolates pws1 **(A)**, pws5 **(B)**, pws8 **(C)** and pws11 **(D)** are closely related to *H. mukohataei* DSM 12286, *H. argentinensis* DSM 12282, *H. vallismortis* ATCC 29715, and *H. volcanii* DS2, respectively.

Solar salterns are not a very rich source of nutrients. Besides this, the extreme living conditions, including near saturation concentration of salts, exposure to UV light, and elevated temperature, require a set of genes to aid adaptation and survival under these harsh conditions. So, to understand the genetic diversity in haloarchaea, we performed pangenome analysis. The pangenome defines the total gene pool of a particular set of genomes. Pangenome analysis divides gene pools into three different sets: core, accessory, and unique genes based on their presence in the single or multiple organisms (Medini et al., 2005; Tettelin et al., 2005; Vernikos et al., 2015). Pangenome analysis suggests either an open or closed pangenome in given genera. Species with an open pangenome have multiple new genes added *per* sequenced genome, and hence it becomes challenging to predict the full pan-genome (Tettelin et al., 2005). On the other hand, in a closed pangenome, only limited new genes are added with the addition of a new genome. Therefore, the theoretical size of the pangenome can be calculated (Vernikos et al., 2015). The haloarchaeal isolates obtained in this study belong to three genera i.e., *Haloarcula, Haloferax*, and *Halomicrobium*. Therefore, we randomly picked one representative strain belonging to genera *Haloferax*, and *Halomicrobium* for whole-genome sequencing. Members of the genus *Haloarcula* are known to harbor *bop*, so we selected two isolates (pws5 and pws8) for whole-genome sequencing. The phylogenetic tree based on 16S rRNA sequences extracted from whole-genomes confirms that pws1, pws5, pws8, and pws11 share 99% sequence identity with *Halomicrobium mukohataei* DSM 12286, *Haloarcula argentinensis DSM 12282, Haloarcula vallismortis* ATCC 29715 and *Haloferax volcanii* DS2, respectively (Figure 3). These results were further confirmed by calculating average nucleotide index (ANI) and digital DNA-DNA hybridization scores where the observed values were higher than the accepted cut-off values (for ANI >95% and DNA-DNA hybridization >91%) for novel species. Hence, all four isolates are novel strains and not novel species.

**Figure 4:**
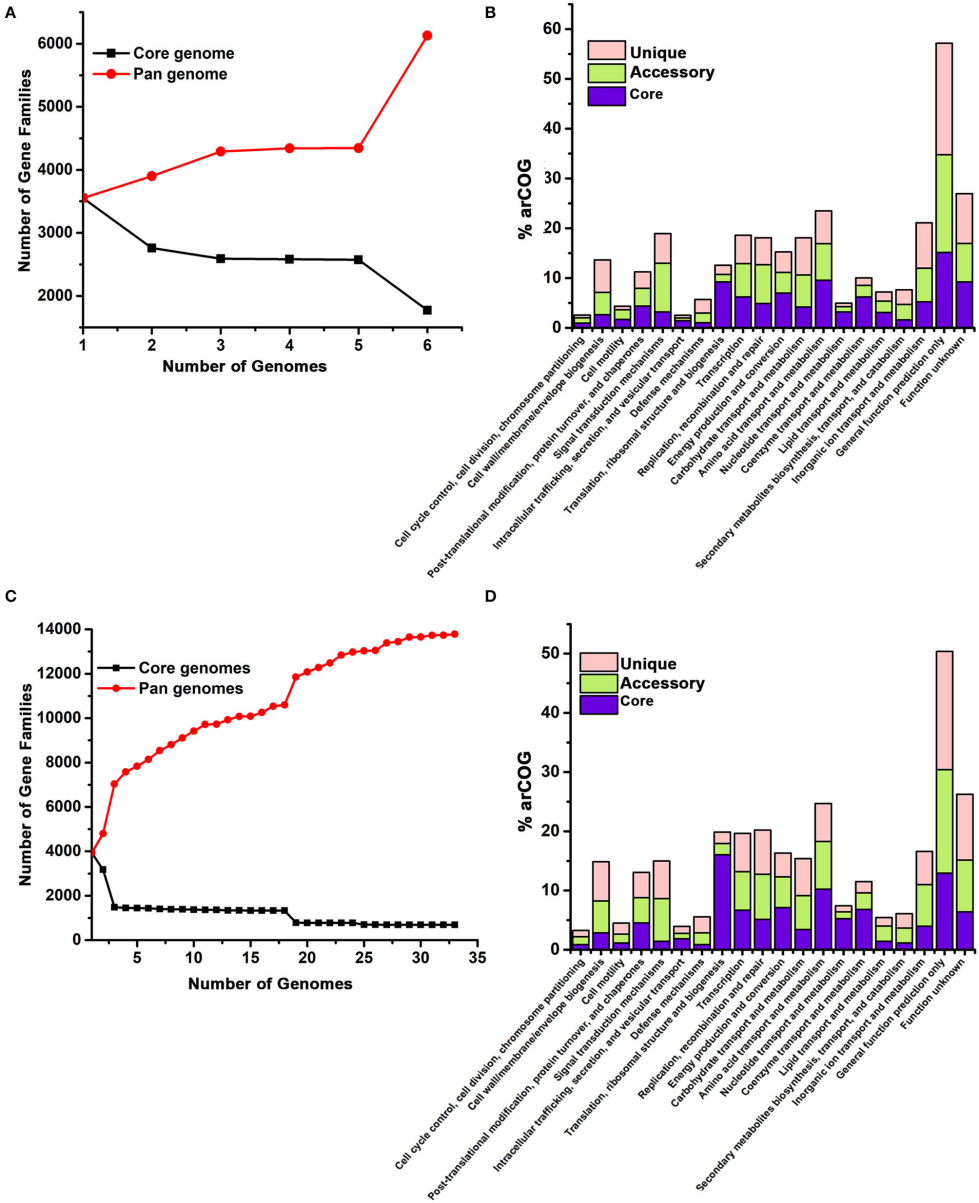
Pangenome analysis of *Halomicrobium* genus **(A,B)** including pws1 strain and *Haloarcula* genus **(C,D)** including pws5 and pws8 strains using the BPGA pipeline. The genome sequences of *Halomicrobium* and *Haloarcula* members were retrieved from NCBI and were further analyzed by the BPGA tool. In both the panels, **(A)** and **(C)** are showing core genome (black) vs. pangenome (red) curves, while panel **(B)** and **(D)** are showing the frequency distribution of core (blue), accessory (olive) and unique genes (pink) within genomes.

The draft genome sequences of pws1, pws5, pws8, and pws11 comprised of 3.39 Mb, 4.0 Mb, 3.9 Mb and 3.67 Mb with 3,722, 4,277, 4,150 and 4,021 annotated coding sequences (CDS). The GC content of pws1, pws5, pws8 and pws11 was 65.7, 61.3, 62.2, and 66.1%, respectively. Whole-genome sequencing data confirmed the presence of *bop* in pws1, pws5, and pws8, and no *bop* was observed in the pws11 genome. This analysis revalidated our initial PCR-based screening results that were designed to identify isolates harboring *bop*. We selected *bop* harboring strains, pws1, pws5, and pws8, for further pangenome analysis (Figure 4). The results of pangenome are described under two following parts:

### Pangenome Analysis of Genus *Halomicrobium*

Both 16S rRNA and whole genomic sequencing results confirmed that pws1 belongs to *Halomicrobium* genus and till now, only five different genomes are known in this genus. We used all available five genomes, including pws1 for pangenome analysis. The distribution of the archaeal clusters of orthologous groups (arCOG) plot of *Halomicrobium* genus (including pws1) suggested that pangenome and core genome contain 6,129 genes and 3,551 genes, respectively. The arCOG distribution plot also suggests that the pangenome is open and increasing with the addition of new genomes that contain several unique genes (Figure 4A). The frequency distribution plot suggests multiple unique and accessory genes insertion including genes coding for cell wall/membrane/envelop biogenesis, signal transduction, replication, recombination, repair, carbohydrate transport, inorganic ion transport, and metabolism-related genes (Figure 4B).

### Pangenome Analysis of Genus *Haloarcula*

// To date, thirty different draft genomes are available under genus *Haloarcula*. We performed a pangenome analysis of pws5 and pws8 with all available thirty *Haloarcula* genomes. For the genus *Haloarcula*, the pangenome and core genome contain 13,782 genes and 690 genes, respectively. The pangenome analysis suggested that similar to *Halomicrobium, Haloarcula* (including pws5 and pws8) pangenome is also open and with the addition of new genomes that contain several unique genes (Figure 4C). They also have similarities in frequency distribution plots such as both genera have multiple insertions of genes coding for cell wall/membrane/envelop biogenesis, signal transduction, replication, recombination, repair, carbohydrate transport, inorganic ion transport, and metabolism-related genes. Additionally, in *Haloarcula* we found multiple other unique and accessory genes, including genes coding for post-translation modifications, chaperons, and cell motility (Figure 4D).

### Expression and Purification of Recombinant Pws5-BR

**Figure 5:**
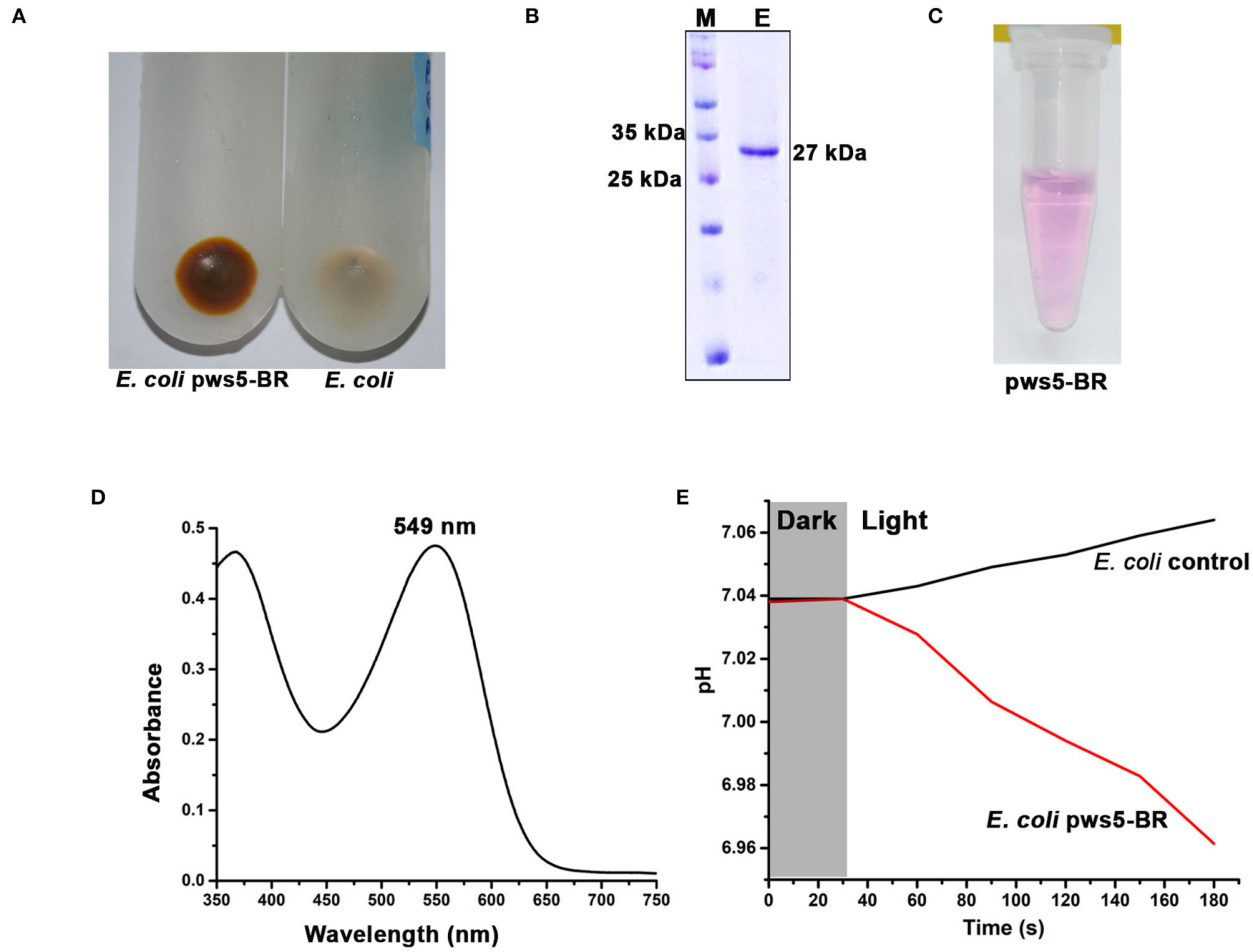
The expression, purification and functional characterization of recombinant pws5-BR. **(A)** The presence of reddish cell pellet suggested the expression of functional pws5-BR. **(B)** The 15% SDS-PAGE showing ~27 kDa purified protein band corresponding to the expected molecular weight of pws5-BR. **(C)** The purple color observed in the purified pws5-BR **(D)** UV-Vis spectra of the purified protein shows 549 nm characteristic BR absorption peak. **(E)** pws5-BR has light-driven proton pumping activity. In pws5-BR expressing cells, pH dropped after exposure to light while there was a marginal increase in pH in control *E. coli* cells suggesting proton pumping activity of pws5-BR.

The full-length sequence of *bop* was extracted from pws5 genome and used for designing gene-specific primers. The *bop* (750 bp) was amplified using gene-specific forward (Bop_full_F) and reverse primers (Bop_full_R). The protein sequence comparison shows that pws5-BR (locus tag -NLV14165.1) shares 92% sequence identity with *H. marismortui* BRI (HmBRI). Based on our previous study (Verma et al., 2019), we chose the pET22b expression vector to yield recombinant protein with C-terminal 6× His-tag for affinity purification. BR is a leaderless membrane protein and putting N-terminal affinity tag may interfere with the membrane localization and protein folding. Protein expression in the presence of trans-retinal yielded red-colored cell pellet suggesting proper membrane integration and functional expression of BR (Figure 5A). The colored cell pellet was further dissolved into DDM (n-Dodecyl-β-D-Maltoside) detergent for BR solubilization. Solubilized protein was then further passed through Ni-NTA beads. Ni-NTA based affinity purification yielded reddish colored protein corresponding to the expected size of ~26 kDa on the 15% SDS-PAGE (Figures 5B,C). UV-Vis spectrum profile gave a characteristic absorbance peak at 549 nm corresponding to retinal bound-BR (Figure 5D). We achieved about 1 mg per liter yield of pws5-BR.

### Light-Driven Proton Pumping Activity of the Recombinant Pws5-BR

To check the functional property of light-driven proton activity in the recombinant pws-BR, we used whole cell-based assay. Briefly, the pws5-BR expressing C43-Rosetta BL21 (DE3) *E. coli* cells and control *E. coli* C43-Rosetta BL21 (DE3) cells (without *pws5* positive clones) were induced with IPTG in the presence of 10 μM retinal. The cells were pelleted, washed, and resuspended in a non-buffered solution, as described in the materials and methods section. The non-buffered solution was used to detect small changes in pH generated by proton pumping activity, which will be otherwise masked by the buffer. The pH probe was dipped in a glass vial having cell suspension and incubated under dark conditions to measure changes in the pH. Under dark conditions, the pH was stable, but when white light was switched on, there was a decrease in the pH compared with the *E. coli* control cells (Figure 5E). This observation is in line with previously published reports (Wang et al., 2003; Kanehara et al., 2017; Verma et al., 2019). The light-induced pH shift was observed due to the proton-pumping activity of the pws5-BR.

### Genetic Organization of *bop* in Haloarchaea

**Figure 6:**
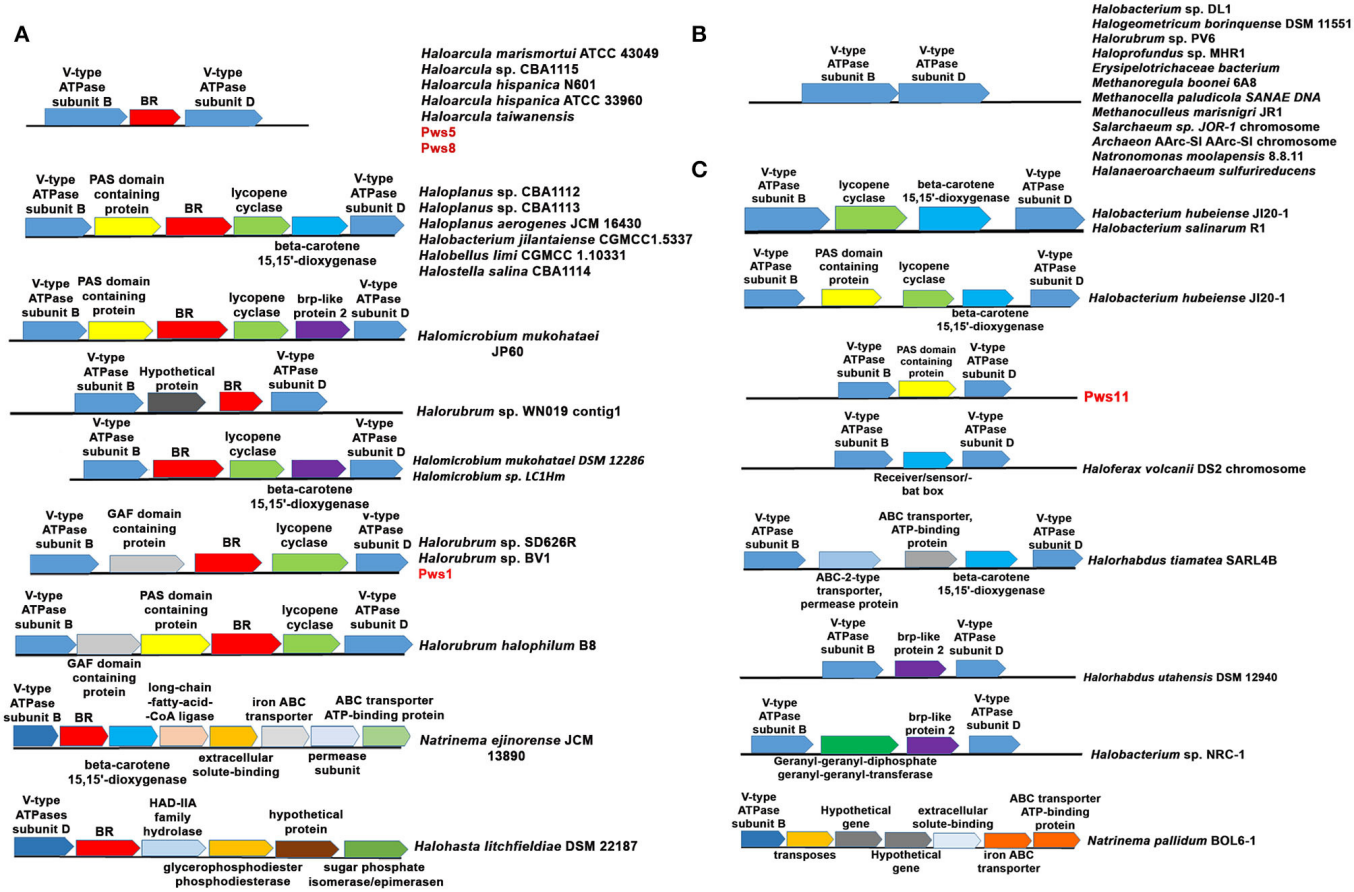
Genetic architecture of *bop* operon in haloarchaea. **(A)** The haloarchaeal strains harboring *bop* and other genes located between the genes coding for B and D subunits of V-type ATPases operon. **(B)** Haloarchaeal members having no additional genes inserted between B and D subunits of V-type ATPases. **(C)** Haloarchaeal members having genes, excluding *bop*, inserted between B and D subunit of V-type ATPases.

For the functional expression of BR and proton-driven ATP generation, several genes are required, including genes involved in retinal biosynthesis, transcription regulation, and V-type ATPases (Sharma et al., 2007). To study the genetic organization of *bop*, we performed analysis of the neighboring genes. In all three isolates, *bop* is located between B and D subunit of V-type ATPases similar to that observed in HmBRI. In the pws1 genome, we additionally found two accessory genes named bacterio-opsin-related protein (*brp*) or GAF domain-containing protein and lycopene cyclase (*crtY*) along with *bop*. In contrast, no additional genes were present in pws5 and pws8 (Figure 6A). The *crtY* and *brp*, along with other enzymes, help in producing retinal from lycopene (Peck et al., 2001; Tarasov et al., 2008). Surprisingly, in pws11, we found only a bacterio-opsin activator protein (*bat*) or Pas domain-containing protein (Montero-Lobato et al., 2018) present in between B and D subunit of V-type ATPases. However, no *bop* was observed in the genome (Figure 6C). The *bat* function as a transcription factor which regulates the *bop* expression (Mirfeizollahi et al., 2019). It will be interesting to study the role of accessory genes in pws1 and pws11 and the presence of *bat* in *bop* deficient strain pws11.

The differences in the genetic organization observed in the region coding for *bop* in pws strains prompted us to perform this analysis across all the sequenced haloarchaeal species. We observed three distinct features in the genes coding for *bop* in *H. salinarum* and *H. marismortui*. (1) In *H. salinarum*, bop operon includes many additional accessory genes that are involved in retinal biosynthesis, BR over-expression, folding and membrane integration. In contrast, in *H. marismortui* HmBRI, only *bop* is present between B and D subunits of V-type ATPases. (2) *H. salinarium bop* has its specific promoter while HmBRI has no separate promoter region and may express with other neighboring genes like V-type ATPases in the predicted operon. (3) In *H. salinarum*, BR over-expression is light-dependent and regulated by the *brz* while HmBRI expresses constitutively and had no effect on expression upon light exposure (Fu et al., 2010).

All these observations suggest that during evolution, HmBRI might have randomly inserted between B and D subunit of V-type ATPases. We also found many other bops but those are not conserved and their location is also not very specific. Therefore, we have not included those set of genes in our analysis. Some of these examples are listed in Supplementary Figure S3. Whole-genome analysis of multiple haloarchaeal species revealed that similar to *H. marismortui* HmBRI, many other haloarchaeal species also have *bop* present in between B and D subunit of V-type ATPases (Figures 6A,B). Similar to pws1, multiple other *bop* related accessory genes were found between the B and D subunits of V-type ATPases in other haloarchaeal species (Figure 6). These *bop* related accessory genes include *crtY, bat*, β-carotene 15, and 15′-dioxygenases (*blh*) genes. In *H. salinarum* NRC-1 and R1 strains V-type ATPases operon, which is distinct from *bop* operon, also have *crtY* and *blh* genes in between B and D subunits (Figure 6B). These are an additional copy of the *crtY* and *blh* genes as a similar set of genes are also present within the *H. salinarum bop* operon. We also found many haloarchaeal strains having probably similar V-type ATPase operon organization like that observed in the pws11 strain where only BR associated (without *bop*) genes are present (Figure 6C). The function of BR associated genes in the absence of rhodopsin is not clear.

In *Natrinema ejinorense* JCM 13890 and *Halohasta litchfieldiae* DSM 22187, *bop* is located close to B subunit. There are many non-BR associated genes, including ABC transporter, iron transporter, extracellular solute binding protein, transposes, and permeases in the vicinity (Figure 6A lower panel). We also did not observe any D subunit coding gene close to B subunit in both of these V-type ATPase operons. Analysis of multiple genomes of different *Halorubrum* strains revealed that different strains of genus *Halorubrum* acquired different sets of genes between B and D subunits of V-type ATPases. For example, *Halorubrum* sp. WN019 has *bop* with an additional hypothetical gene, *Halorubrum* sp. SD616R have *bop* with GAF domain protein gene and *CrtY, Halorubrum halophilum* B8 has GAF domain protein, *CrtY* and *bat* (Figure 6A). Similarly two different type of *bop* operon found in different *Halomicrobium* strains (Figure 6A).

We also observed that in many haloarchaeal strains, no additional genes were present between B and D subunit of V-type ATPase (Figure 6B). In many cases, some genes were present however, no *bop* was observed (Figure 6C). These findings further suggest that *bop* and other genes might have probably randomly inserted between B and D subunits of V-type ATPases. The differences observed in the genetic organization of genes in the probable *bop* operons raises an important question about the role of different accessory genes in *bop* function and expression. Multiple sequence alignment of different haloarchaeal V-type ATPases operon sequences suggested that no insertions were observed between the B and D subunits coding regions (highlighted with an arrow in Supplementary Figure S4). We also searched for recombination hotspot regions and transposable element insertion sequences in the V-type ATPases, but we did not observe any such conserved sequences.

## Discussion

Haloarchaea have adapted to survive and grow under harsh conditions like high salt concentration, high temperature, high ionic stresses, UV light exposure, alkaline pH and nutrient limitation conditions. Several genes coding for different enzymes, proteins, and biomolecules such as BR and carotenoids facilitate haloarchaeal survival in such harsh environmental conditions. The main objective of this study was to explore the Indian solar saltern to study diversity and isolate new haloarchaeal strains harboring natural BR variants. We successfully isolated 12 haloarchaeal strains from the high salinity environment. Out of twelve, 11 were confirmed as BR harboring haloarchaeal strains using PCR-based screening assay. We also successfully cloned, expressed and purified one of the recombinant BR (pws5-BR) using *E. coli* as an expression host. It is challenging to express BR in functional form in *E. coli*. However, we were able to express pws5-BR using the strategy adopted in our previous study (Verma et al., 2019). Multiple sequencing alignment analysis suggested that pws5-BR shares 94% sequence identity with HmBRI. Considering the potential of BRs in several commercial applications, these high yielding BRs may be explored to exploit them as a substitute for *H. salinarum* BR. This will help in bringing down the cost of recombinant BR as the cost of natural BR is one of the major bottlenecks in commercializing these technologies. However, these recombinant BRs are not as stable as *H. salinarum* BR (Seyedkarimi et al., 2015; Verma et al., 2019). In the future, it will be desirable to work on improving the thermal stability of these BRs to make technologies based on these proteins a reality.

To gain insights into the genetic diversity in the isolated strains whole-genome sequencing followed by pangenome analysis were performed for pws1, pws5 and pws8 strains. The number of identified coding DNA sequences (CDS) ranged from 3,722 to 4,277. The pws5 genome having the largest genome size ~4.0 Mb with 4,277 CDS, while pws1 has the smallest genome size ~3.4 Mb with 3,722 CDS. Pangenome analysis of the protein-coding complements of pws1, pws5, and pws8 revealed that several functional genes were added into different categories, including metabolism and transport of amino acids, inorganic ions, carbohydrates, and secondary metabolites as well as genes involved in signal transduction, translation and cell division. The arCOG distribution plot of pws1, pws5, and pws8 with their respected genus suggested that they have open pangenome which is increasing with the addition of new genome sequences. So, it will be interesting to sequence more isolates to study the genetic diversity in these genera, which may help us in understanding the genes involved in growth and adaptation.

Multiple BR operon analysis of different haloarchaeal genomes (including pws isolates) revealed that in *bop*, if present, is mainly present between the genes coding for B and D subunits of V-type ATPases however, in some cases, *bop* alone or along with some *bop* accessory genes is present in other locations as well. We also attempted to purify BR from the three pws isolates, but we were not successful (data not shown). In previous studies, it has been shown that BR is constitutively expressed in *H. marismortui* (Fu et al., 2010). Our data analysis also suggests, unlike *H. salinarum* the presence of light-inducible transcription regulator is absent in these isolates and many other haloarchaeal species reported in the past. This may be one of the probable reasons for poor or less expression of BR in these haloarchaeal isolates.

To summarize, this study reports four new haloarchaeal strains, isolated from the Indian solar salterns. We also report the differences observed in the *bop* operon/genetic architecture in different haloarchaeal species. We are also successful in purifying functional recombinant pws5-BR using *E. coli* as an expression host. This finding also highlights the presence of several industrially important enzymes and metabolites present in these haloarchaeal isolates.

## Materials and Methods

### Isolation of Halophilic Archaea and Analysis of Samples

Solar salterns water samples were collected in bottles (500 ml each) from solar saltern situated on the ECR Highway of Kottakuppam, near to Puducherry, Tamil Nadu India (11°59′17.39″ N and 79°50′17.39″ E) and haloarchaeal species were isolating using membrane filtration technique as suggested by Montalvo-Rodriguez et al. (1998); and Elevi et al. (2004). The samples were named as pws1 to pws12, respectively. Enrichment technique was employed for the isolation of halophilic archaea. All pws samples were initially filtered using vacuum filtration techniques through 0.45-micron membrane filters, and the filtered porous membranes were transferred to halobacterium agar media (HB media) (HiMedia) and incubated for 2–3 weeks at 37°C. The filter pieces containing pink-red patches were further transferred to sterile HB liquid medium containing 250 g/l NaCl, 3 g/l, trisodium citrate, 20 g/l MgSO4. 7H20, 2 g/l KCl and 10 g/l Oxoid peptone. After 1 week of incubation at 37°C, 200 rpm red-pink color fermentation broth indicates the different carotenoid producing haloarchaeal strains. Repeated subculturing for 3–4 times in HB media yielded pure single colony red/pink pigmented colonies on the agar plates. Each colony was separately inoculated into 20 ml HB medium and incubated at 37°C with 200 rpm for genomic DNA isolation to study 16S rRNA taxonomic diversity and *bop* PCR amplification studies.

### Genomic DNA Isolation and 16S rRNA Sequencing

Genomic DNA extractions of all twelve strains were performed by Zymo research genomic DNA isolation kit (Cat No. D6105). The partial 16S rRNA sequences were amplified using standard primers 21F and 1453R (Supplementary Table 1). The other 16S rRNA sequences used in the comparative analysis were taken from the NCBI database (https://www.ncbi.nlm.nih.gov/). The amplified partial 16S rRNA gene sequences were further used for taxonomic identification using the EzTaxon server (Chun et al., 2007).

### Phylogenetic Tree Construction

The 16S rRNA sequences used for phylogenetic analysis were aligned using program MUSCLE (v3.8.31) with default settings (Edgar, 2004). Ambiguous regions i.e., poorly aligned or regions containing gaps were removed from the aligned sequences using Gblocks (v0.91b) (Castresana, 2000). The phylogenetic trees were reconstructed with PhyML program (v3.1/3.0 aLRT) using the maximum likelihood method (Guindon and Gascuel, 2003). The default substitution model was selected assuming an estimated proportion of invariant sites (of 0.748, 0.761, 0.840, and 0.940 for pws1, pws5, pws8, and pws11) and 4 gamma-distributed rate categories to account for rate heterogeneity across sites. The gamma shape parameter was estimated directly from the data. Bootstrapping method (100 bootstrap replicates) was used to assess the reliability of the internal branch (Anisimova and Gascuel, 2006). Graphical representation of the phylogenetic trees were prepared using iTOL (Letunic and Bork, 2019).

### Transmission Electron Microscopy

Aliquots of 50 μl from the haloarchaeal grown cultures for 4–5 days at 37°C with constant shaking at 200 rpm were added on a carbon-coated 300-mesh copper grid (Polysciences Asia Pacific, Taiwan). The excess sample was blotted, followed by air drying. The grids were further imaged using a JEM 2100 electron microscope (JEOL), operated at 200 keV.

### Carotenoid Isolation

Carotenoids were isolated following a acetone/methanol extraction method (Yatsunami et al., 2014). Briefly, 20 ml of grown culture were pelleted at 18,000 × g for 30 min. Supernatants were discarded and colored pellets were dissolved in 40 mL of acetone and methanol mixture prepared with 7:3 ratio. The samples were incubated at room temperature for 30 min in dark and again centrifuged at 18,000 × g for 30 min. Final colored supernatants were collected and speedvac at 37°C to evaporate methanol and acetone. Reddish-pink colored pellets were later dissolved in 50 μl of methanol. UV-visible spectra of extracted carotenoid solutions were recorded from 200 to 700 nm range using a UV-Vis spectrophotometer (CECIL CE7500 spectrophotometer).

### Screening of *bop* From pws Isolates

The isolated strains were screened for the presence of *bop* using a PCR-based screening assay. Multiple sequence alignment was performed to identify highly conserved regions in *bop* and design a set of degenerate primers against conserved regions (Supplementary Table 1). Isolated whole-genome DNA samples were subjected to PCR-based amplification using these conserved primers (DegF and DrgR). The expected size of PCR product amplified using these primers is around 450 bp.

### Cloning, Expression, and Purification of BRs

The full-length pws5-*bop* was amplified by gene-specific primers named Bop_full_F and Bop_full_R (Supplementary Table 1). The amplified fragments were further digested with NheI-XhoI restriction enzymes and cloned into pET22b vector for C-terminal 6× his-tag. The pws-5 *bop* has several rare codons. So, to aid expression we used C43-Rosetta BL21 (DE3) *E. coli* cells as reported in our earlier study (Verma et al., 2020). *E. coli* C43-Rosetta BL21 (DE3) cells are C43 (DE3) cells harboring the pRARE plasmid isolated from Rosetta (DE3) cells. The growing culture was induced at OD 0.6 with 0.5 mM IPTG and 5–10 μM retinal (Sigma-Aldrich). 100% ethanol was used to prepare trans-retinal stocks. The culture was incubated for 4 h in an incubator shaker at 37°C and kept shaking at 200 rpm. The culture was centrifuged at 9,000 × g for 15 min and resuspended in lysis buffer A (20 mM Tris, pH 7.5 and 4 M NaCl). The cells were sonicated and centrifuged at 18,000 x × g for 60 min to obtain membrane fraction in the pellet. The colored pellet was further resuspended in lysis buffer A having 0.2% DDM (Anatrace, USA) and incubated overnight. The role of DDM detergent was to extract BR from the insoluble membrane fraction. The soluble colored fraction was incubated with Ni-NTA resin for 1 h to facilitate binding, and the colored protein was eluted using elution buffer E (20 mM Tris pH 7.5, 0.02% DDM and 4 M NaCl, with 500 mM imidazole). The purified protein was concentrated and dialyzed against buffer composed of 20 mM Tris pH 7.5, 0.02% DDM and 4 M NaCl to remove imidazole.

### BR Spectral Analysis and Proton Pumping Activity

The 10 μM of purified pws5-BR was used to perform UV-Vis spectral scanning (200 to 800 nm) using CECIL CE7500 spectrophotometer. The light-based proton pumping assays were performed as described earlier by Wang et al. (2003). Briefly, 20 ml culture of BR expressing C43-Rosetta BL21 (DE3) *E. coli* cells were centrifuged at 15,000 × g at 4°C, washed and resuspended in a non-buffered solution (10 mM NaCl, 10 mM MgSO4 and 100 mM CaCl_2_). For the proton pumping experiment, OD_600_ was adjusted to 2.0 in dark. The experiment was started by illuminating the cells with the continuous high-intensity white light source for 120s. The change in pH was measured by using a JENWAY 3510 pH meter.

### Genome Sequencing, Assembly, and Data Submission

Four pws isolates named pws1, pws5, pws8, and pws11 were subjected for whole-genome sequencing using Illumina NextSeq 500 and assembled using the CLC NGS Cell 9.0. The whole-genome sequencing was performed by Bionivid Technology Pvt Ltd, India. The whole-genome sequencing data of pws1, pws5, pws8, and pws11 data were submitted to the NCBI database under accession numbers WOYG00000000, WOWA00000000, WOWB00000000, and WOWC00000000, respectively. The draft genomes were annotated using the RAST pipeline online server (Aziz et al., 2008). Genome comparison and pangenome analysis were carried out by using the EzTaxon and BPGA pipeline, respectively (Chun et al., 2007; Chaudhari et al., 2016).

## Data Availability Statement

The datasets generated for this study can be found in online repositories. The names of the repository/repositories and accession number(s) can be found in the article/Supplementary material.

## Author Contributions

KT, SP, and DV conceived the study. KT and SP coordinated the study. SP and BS provided the strains. KT and DV designed experiments, analyzed data, and wrote the paper with inputs from other coauthors. DV, LS, CC, and CS performed experiments. All authors reviewed the results and approved the final version of the manuscript. All authors contributed to the article and approved the submitted version.

## Conflict of Interest

The authors declare that the research was conducted in the absence of any commercial or financial relationships that could be construed as a potential conflict of interest.
