# Corrigendum: Isolation and Taxonomic Characterization of Novel Haloarchaeal Isolates From Indian Solar Saltern: A Brief Review on Distribution of Bacteriorhodopsins and V-Type ATPases in Haloarchaea

**DOI:** 10.3389/fmicb.2021.713942

**Published:** 2021-06-28

**Authors:** Dipesh Kumar Verma, Chetna Chaudhary, Latika Singh, Chandni Sidhu, Busi Siddhardha, Senthil E. Prasad, Krishan Gopal Thakur

**Affiliations:** ^1^Structural Biology Laboratory, G. N. Ramachandran Protein Centre, Council of Scientific and Industrial Research-Institute of Microbial Technology (CSIR-IMTECH), Chandigarh, India; ^2^MTCC-Microbial Type Culture Collection & Gene Bank, Council of Scientific and Industrial Research Institute of Microbial Technology (CSIR-IMTECH), Chandigarh, India; ^3^Department of Microbiology, School of Life Sciences, Pondicherry University, Puducherry, India; ^4^Biochemical Engineering Research and Process Development Centre, Council of Scientific and Industrial Research-Institute of Microbial Technology (CSIR-IMTECH), Chandigarh, India

**Keywords:** haloarchaea, bacteriorhodopsin, pangenome, carotenoids, taxonomy

In the original article, there was an inadvertent mistake in the preparation of Figure 1. While preparing the figure, the transmission electron microscopy (TEM) micrograph panel for pws12 was inadvertently selected from the panel of TEM micrographs collected for pws4. The corrected Figure 1 appears below.

**Figure 1:**
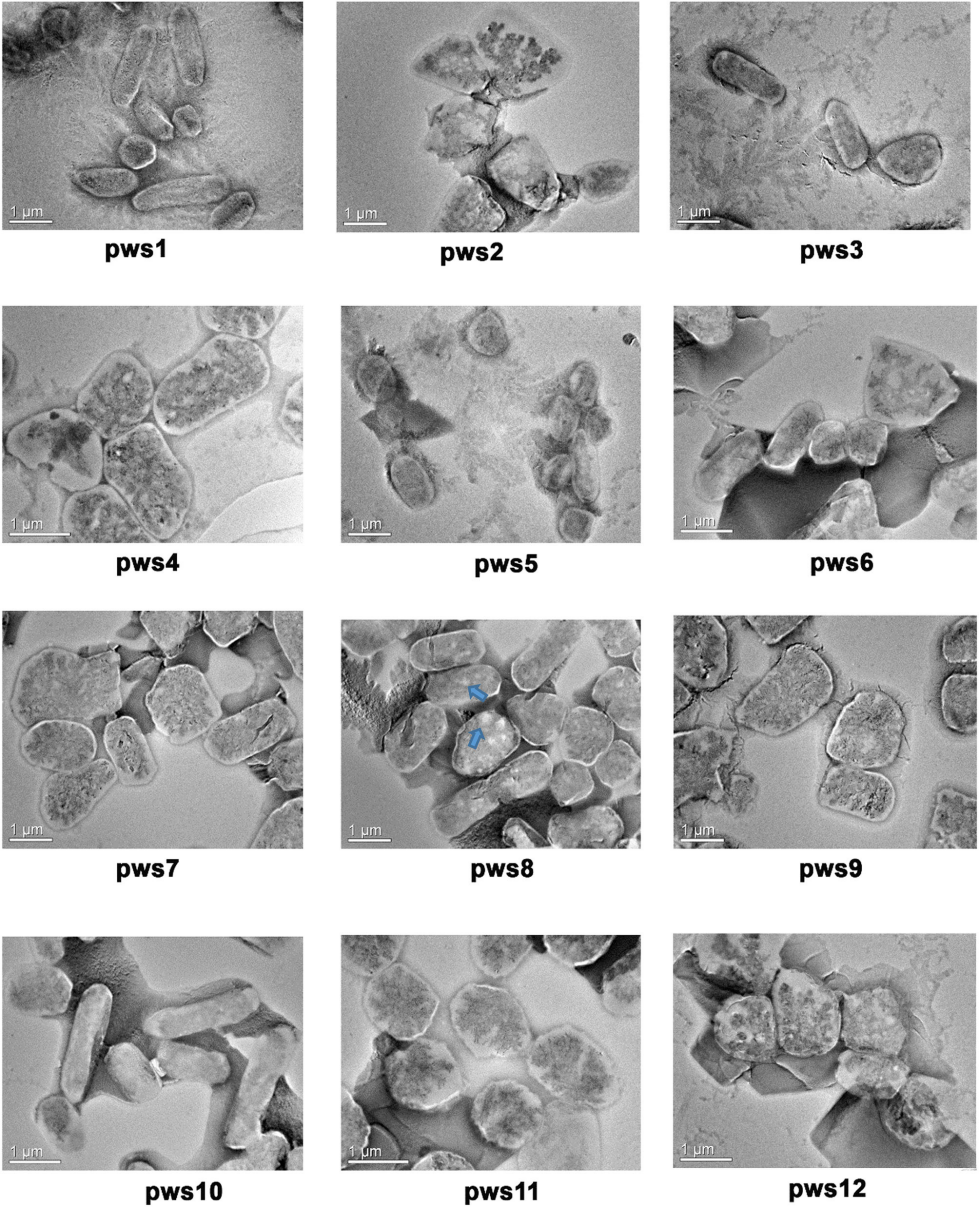
Transmission electron microscopy images of pws isolates. TEM images reveal polymorphic morphology in the haloarchaeal isolates. Blue arrows indicate the presence of gas vacuoles.

The authors apologize for this error and state that this does not change the scientific conclusions of the article in any way. The original article has been updated.

