# Supplementary material for: Isolation and Taxonomic Characterization of Novel Haloarchaeal Isolates From Indian Solar Saltern: A Brief Review on Distribution of Bacteriorhodopsins and V-Type ATPases in Haloarchaea
Source: Front Microbiol. 2020 Dec 9;11:554927. doi: 10.3389/fmicb.2020.554927 (PMC7755889; doi:10.3389/fmicb.2020.554927)
Supplement: Supplementary file 1 [file Data_Sheet_1.PDF]

Supplementary Information

Isolation and taxonomic characterization of novel phototropic Haloarchaeal isolates from Indian solar saltern: A brief review on distribution of bacteriorhodopsins (BR1 and V-type ATPases in Haloarchaea

Dipesh Kumar Verma[1], Chetna Chaudhary[1], Latika Singh[1], Chandni Sidhu[2], Busi Siddhardha[4], Senthil E. Prasad[3#,] and Krishan Gopal Thakur[1#]

[1]G. N. Ramachandran Protein Centre, Structural Biology Laboratory, Council of Scientific and Industrial Research-Institute of Microbial Technology (CSIR-IMTECH), Chandigarh-160036, India.

[2]MTCC-Microbial Type Culture Collection & Gene Bank, CSIR-Institute of Microbial Technology, Chandigarh-160036, India.

[3]Biochemical Engineering Research and Process Development Centre, Council of Scientific and Industrial Research-Institute of Microbial Technology (CSIR-IMTECH), Chandigarh-160036, India.

[4] Department of Microbiology, School of Life Sciences, Pondicherry University, Puducherry - 605008, India

Correspondence

[#]Krishan Gopal Thakur
Email: krishang@imtech.res.in
+91 8427717723

[#]Senthil E. Prasad
Email: esprasad@imtech.res.in
+91 9316166198

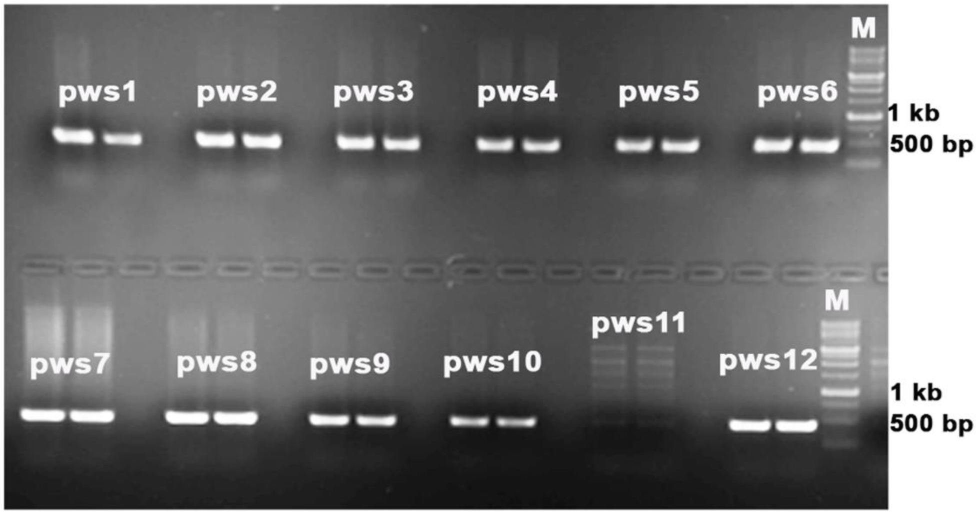

Figure S1. The bop screening in pws isolates. PCR-based screening for the presence of the *bop*. The amplified PCR products were resolved on 1% agarose gel. The presence of a PCR product (~450 bp) suggests the presence of the *bop* in a given isolate. The experiments were performed in duplicates.

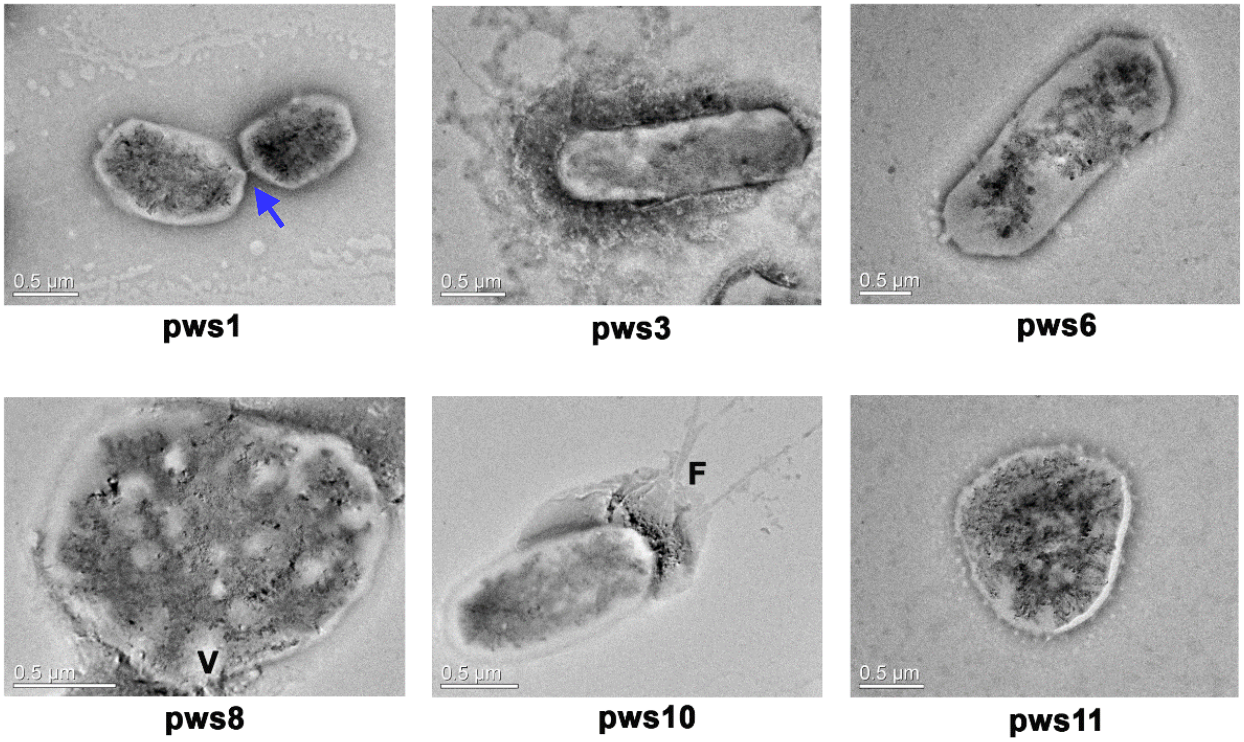

Figure S2. TEM images showing distinctive features observed in the pws isolates. In pws1 pilus like structure connecting two cells (blue arrow) was observed, both pws3 and pws6 showing thick extracellular material, pws8 is highly vacuolated among the isolates, long multiple cellular appendages were observed at one pole in pws10 isolate and multiple secreted vesicle like structures were observed in pws11.

*F – flagella like extracellular appendage

*V – vacuole

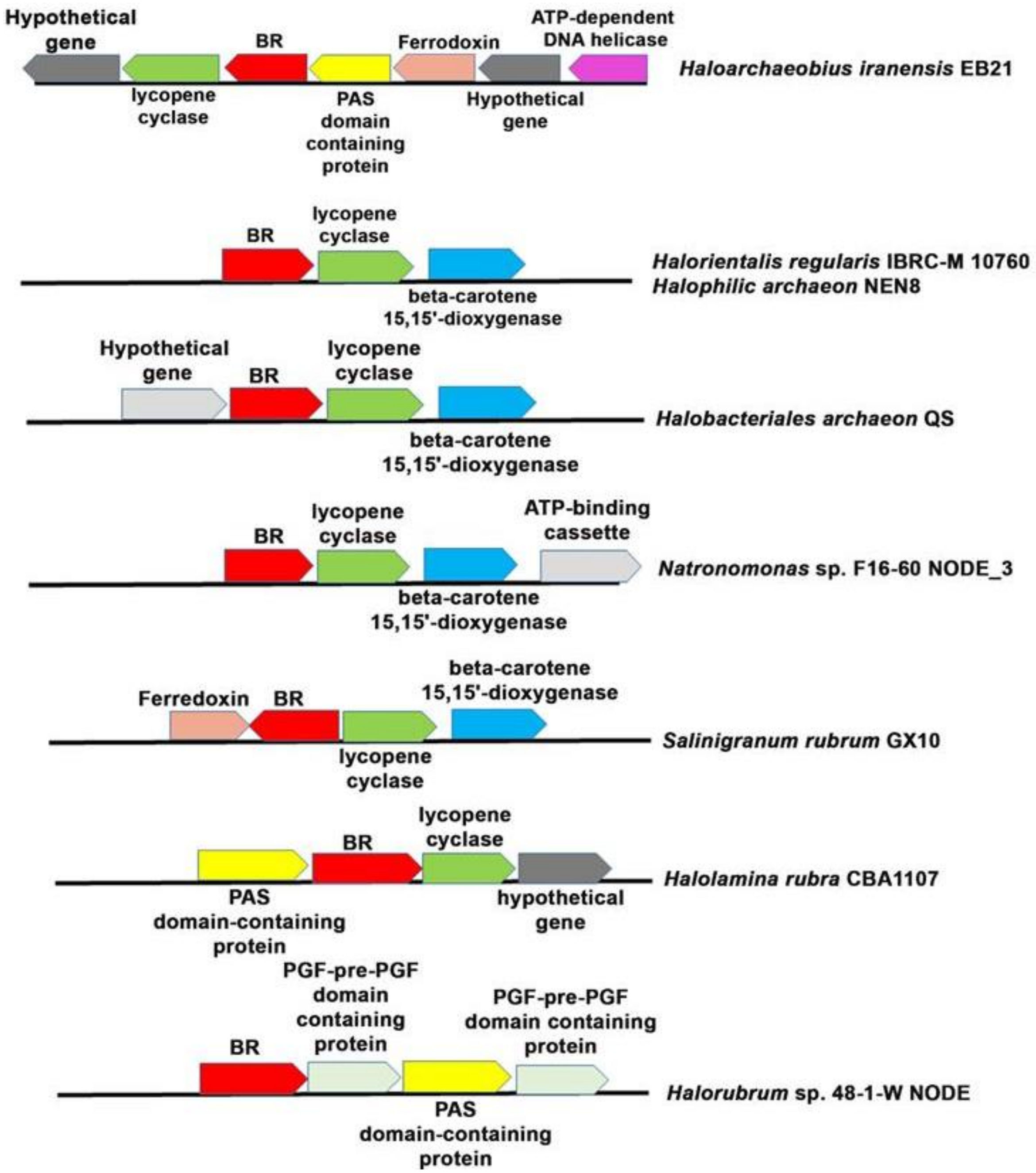

Figure S3. The presence of *bop* in the genomic locations other than V-type ATPases operon in some haloarchaeal species.

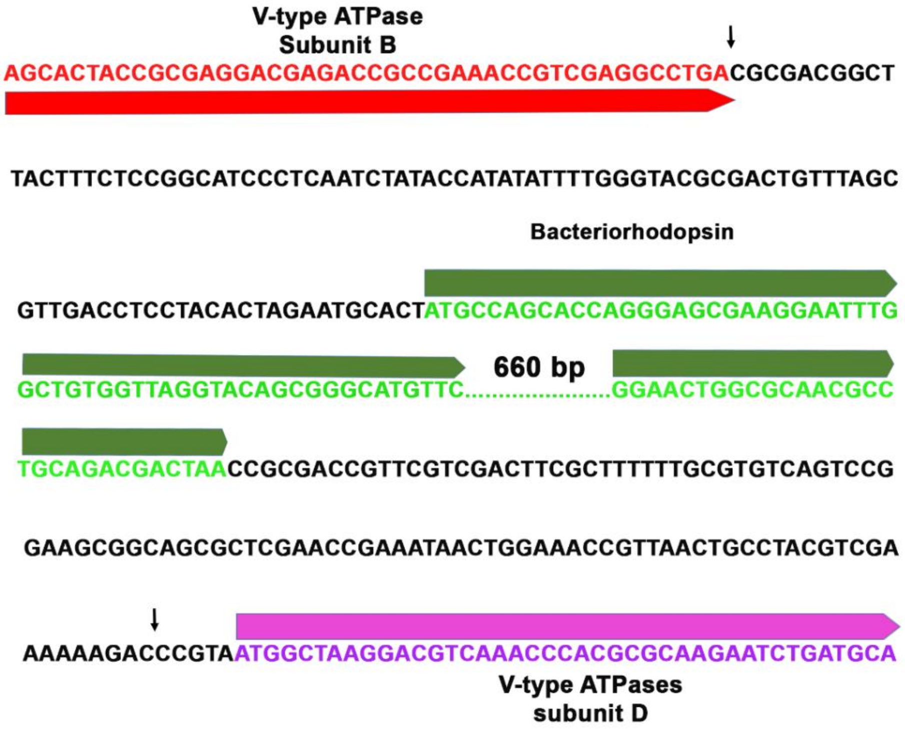

Figure S4. Schematic representation of HmBRI operon. The genes including *bop* is inserted between the B and D subunits (highlighted by arrow) in several haloarchaeal species.

Supplementary Table 1. A list of primers used in the study.

**Name**	**Primer sequence**
21F	TCCGGTTGATCCYGCCGG
1453R	GGGCYGCACGCGYRCTACA
DegF	GACTGGTTGTTYATVACGCC
DegR	AASCCGAAGCCGAYCTTBGC
Bop_full_F	ACCGAAGCTAGCTTCCTCGGCATGCTCTACTTCATC
Bop_full_R	CTTGCTCGAGGTCGTCTGC AGGCGTTG CGCC
